# Synthesis and Biological Evaluation of Bicyclic Pyrazolines with Promising Antimicrobial Activities

**DOI:** 10.1002/cmdc.202500144

**Published:** 2025-05-27

**Authors:** Debora Caviglia, Anna Maria Schito, Susanna Penco, Chiara Brullo, Marcus Baumann

**Affiliations:** ^1^ Department of Surgical Sciences and Integrated Diagnostics University of Genova Viale Benedetto XV, 6 16132 Genoa Italy; ^2^ Department of Pharmacy Section of Medicinal Chemistry University of Genova Viale Benedetto XV, 3 16132 Genoa Italy; ^3^ Department of Experimental Medicine University of Genova Via L.B. Alberti, 2 16132 Genoa Italy; ^4^ School of Chemistry University College Dublin Science Centre South Belfield Dublin Ireland

**Keywords:** antimicrobial resistances, bacteriostatic properties, drug‐likeness properties, flow syntheses, photo‐click reactions, pyrazolines

## Abstract

The efficient photochemical synthesis of bicyclic pyrazolines using flow technology as well as the subsequent antimicrobial evaluation of these scaffolds is reported. Low minimal inhibitory concentration values of 0.5–4 μg mL^−1^ are found against a series of multidrug resistant bacterial strains including different *Staphylococcus* and *Enterococcus* genera species. The lead compound, which is decorated by a halogenated aryl ring system, appears to be bacteriostatic and shows excellent physicochemical and pharmacokinetic properties. Due to low levels of predicted toxicity combined with a high level of drug‐likeness, these bicyclic pyrazolines therefore are promising candidates for further studies as antibacterial species.

## Graphical abstract

Continuous flow technology is exploited for the generation of bicyclic pyrazolines via photo‐click reactions. These heterocyclic species contain a valuable 3D scaffold along with a polar succinimide moiety. Subsequent antimicrobial testing reveals that halogenated moieties such as chlorine atoms and trifluoromethoxy groups enhance the potency of these bacteriostatic compounds reaching low minimal inhibitory concentration values (0.5–4 μg mL^−1^).

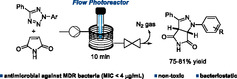

## 1 Introduction

**Figure 1:**
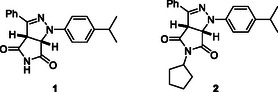
Structures of hit compound **1** and its inactive derivative **2**.

Microbial resistance remains one of the most significant health challenges of the 21st century. While mortality rates differ significantly based on region and age group, estimates suggest that almost 40 million deaths will be caused by resistance to microbes in the next 25 years.^[^ ^1^ ^]^ Due to resistance of the most used conventional antibiotics, the Infectious Diseases Society of America (IDSA) has recognized the bacteria of the ESKAPE group (*Enterococcus faecium*, *Staphylococcus aureus*, *Klebsiella pneumonia, Acinetobacter baumannii*, *Pseudomonas aeruginosa,* *Enterobacter* species) as the most dangerous pathogens.^[^ ^1^ ^]^ Consequently, significant efforts are underway targeting the discovery of new chemical entities with antimicrobial properties, including both de novo synthesis of suitable candidates and repurposing strategies of existing drugs.^[^ ^2^ ^]^ Recently, we reported on the synthesis and screening of a small collection of indazoles and related pyrazoles and pyrazolines, and their study in the context of their antimicrobial properties.^[^ ^3^ ^]^ This showed a hit for a novel bicyclic pyrazoline scaffold bearing a succinimide moiety (i.e., **1**, **Figure** **1**). In detail, pyrazoline **1** rendered excellent potency (minimal inhibitory concentration, MIC) values of 4 μg mL^−1^ against the most relevant clinical species of the genus *Staphylococcus*, such as *S. aureus* and *S. epidermidis*, and of the genus *Enterococcus*, such as *E. faecalis* and *E. faecium*, including several drug‐resistant variants.^[^ ^3^ ^]^ At the same time, it was found that the substitution of the succinimide nitrogen atom (i.e., compound **2**) reduced activity significantly, highlighting the relevance of this moiety, which may indicate the value of this hydrophilic group for increased solubility and H bonding.

**Scheme 1:**
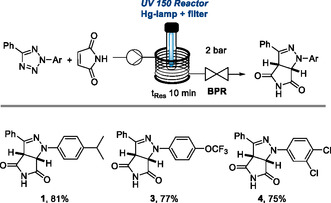
Continuous photochemical synthesis of pyrazolines **1**, **3,** and **4**.

To better understand how general this new scaffold is in imparting antimicrobial activity, we set out to generate a small set of related bicyclic pyrazolines retaining the free NH succinimide and varying the substitution on the *N*‐arylated ring. Specifically, electron withdrawing groups such as a trifluoromethoxy substituent and halogen atoms (compounds **3** and **4**, **Scheme** **1**) were introduced. Pyrazolines **3** and **4** were therefore selected for detailed antimicrobial studies, in analogy to studies performed for hit compound **1**, revealing new insights into the importance of halogenation patterns on the aryl groups as well as the likely mode of action of these species toward different drug‐resistant pathogens.

## 2 Results and Discussion

### 2.1 Synthesis of Pyrazolines

The synthesis of the bicyclic pyrazoline scaffold exploited a crucial photochemical click reaction between aryl tetrazoles and maleimide as key building blocks. The aryl tetrazoles can be generated via a two‐step sequence from substituted anilines via diazotization and a subsequent oxidative cyclization with benzamidine hydrochloride (see Supporting Information for details).^[^ ^4^ ^]^ The following photochemical click reaction^[^ ^5^ ^]^ is an attractive approach facilitating the preparation of small libraries due to its modular nature which allows for simple variation on the aryl moieties present. As this reaction requires the use of high‐energy light to trigger the fragmentation of the tetrazole into a nitrile imine dipole and nitrogen gas prior to the formation of the key pyrazoline via a dipolar cycloaddition, a continuous flow reactor system was exploited. In the context of photochemistry, flow reactors provide several advantages over analogous batch processing due to more uniform irradiation of the reactor coil inside a temperature‐controlled casing.^[^ ^6^ ^]^ This leads to high photon flux and spatiotemporal resolution ensuring that over‐irradiation and photodegradation phenomena are minimized. Moreover, short residence times and higher substrate concentrations are frequently viable when using flow photochemistry, which leads to higher productivity and the ability to scale up reactions by operating the setup for longer periods of time under steady‐state conditions.^[^ ^7^ ^]^ For the generation of bicyclic pyrazolines, a Vapourtec E‐Series flow reactor was used in combination with a filtered medium‐pressure Hg lamp. As shown in Scheme 1, a stock solution containing the aryl tetrazole (100 mM, MeCN) and maleimide (1.2 equiv.) was pumped through the irradiated reactor coil (perfluoroalkoxy alkane, 10 mL, 1/8‐inch diameter) and the reaction mixture was collected after passing a back‐pressure regulator (set to 2 bar) which allowed the controlled release of the nitrogen gas by‐product. The desired pyrazoline products (**1**, **3**, **4**) were generated within short residence times (*t*_Res_ = 10 min) and high yields allowing for the preparation of gram quantities of these targets for subsequent antimicrobial testing.

### 2.2 Antimicrobial Evaluation

The antibacterial activity of compounds **3** and **4** was tested by calculating their MIC values on a total of thirty‐six clinical strains, most of which were multidrug resistant (MDR) toward current antibiotics. Thirty‐three of these were Gram‐positive organisms which included twenty‐five isolates of the genus *Staphylococcus*. In detail, five strains were *Staphylococcus aureus*, four of which were resistant to methicillin (MRSA), five were *Staphylococcus epidermidis*, four of which were resistant to methicillin (MRSE), and fifteen more isolates were related to eight different species of the same genus. The remaining eight Gram‐positive isolates were four *Enterococcus* *faecalis* and four *Enterococcus faecium* strains, variously resistant to vancomycin (VRE) and teicoplanin. In addition, three MDR Gram‐negative strains were also tested: *Escherichia coli*, *Pseudomonas aeruginosa*, and *Klebsiella pneumoniae*.

In analogy to the previous hit compound **1**, the novel compounds **3** and **4** were considered poorly active when MIC values above 128 μg mL^−1^ were observed, as in the case of the Gram‐negative isolates tested here. On the contrary, the activity of compounds **3** and **4** was found to be excellent on all Gram‐positive strains tested, including the MDR strains. In **Table** **1**, MIC values of compounds **3** and **4** against *S. aureus*, *S. epidermidis*, *E. faecalis*, and *E. faecium* strains are reported, whereas in **Table** **2** activity against additional *Staphylococcus* species (fifteen isolates) is shown.

**Table 1:** MIC values of compounds **3** and 4 against *S. aureus*, *S. epidermidis*, *E. faecalis*, and *E. faecium* strains and those of reference antibiotics expressed in μg mL^−1^; experiments were carried out in triplicate.

Strains used	MIC [μg mL^−1^]		
	3	4	Reference antibiotics
*S. aureus* 17a)	4	2	256 (O)
*S. aureus* 18a)	4	2	512 (O)
*S. aureus* 195a)	4	2	256 (O)
*S. aureus* 197	4	2	1 (O)
*S. aureus* Aa)	4	2	256 (O)
*S. epidermidis 22* a)		2	256 (O)
*S. epidermidis 171* a)	4	2	128 (O)
*S. epidermidis 180*	4	2	0.25 (O)
*S. epidermidis 181* b)	4	2	128 (O)
*S. epidermidis 2R* a)	4	2	256 (O)
*E. faecalis 1* c)	8	4	256 (V), 64 (T)
*E. faecalis 79* c), d)	8	4	256 (V), 128 (T)
*E. faecalis 50* d)	8	4	32 (V), 0.5 (T)
*E. faecalis 365* d)	8	4	32 (V), 1 (T)
*E. faecium 51* c), d)	8	4	128 (V), 64 (T)
*E. faecium 300* d)	16	4	64 (V), 1 (T)
*E. faecium 365* d)	8	4	32 (V), 0.5 (T)
*E. faecium 503*	16	4	0.5 (V), 1 (T)

a) Resistance toward methicillin.

b) Resistance to methicillin and linezolid.

c) Resistance to teicoplanin. Reference antibiotics were oxacillin (O), vancomycin (V), and teicoplanin (T). Gram‐negative strains, for whom MIC values were greater than 128 μg mL^−1^, were not reported.

d) Resistance to vancomycin.

**Table 2:** MIC values against additional *Staphylococcus* species of compounds **3** and **4** and reference antibiotic oxacillin (O), obtained from experiments carried out in triplicate, expressed as μg mL^−1^.

Strains used	3	4	O
*S. auricularis 136* a)	0.5	0.5	16
*S. capitis 71* a)	4	2	64
*S. capitis 72* a)	2	1	64
*S. capitis 121*	4	1	0.25
*S. haemolyticus 115* a)	4	2	64
*S. haemolyticus 193* a)	4	2	16
*S. haemolyticus 174*	4	1	0.25
*S. hominis 124* a)	4	2	16
*S. hominis 125* a)	4	1	64
*S. lugdunensis 96*	4	2	0.5
*S. lugdunensis 137* a)	4	1	16
*S. saprophyticus 41*	4	2	0.5
*S. simulans 94* a)	4	2	16
*S. simulans 163* a)	4	2	16
*S. warneri 74* a ^)^	4	1	64

a) Resistance to methicillin.

The data from these studies show that in most of the selected isolates the new pyrazolines **3** and **4** are characterized by MIC values lower than those of the initial hit compound **1** (0.5–4 vs 4 μg mL^−1^ of compound **1**),^[^ ^3^ ^]^ confirming that the introduction of an electron withdrawing group on the phenyl ring is beneficial for antimicrobial activity. Particularly compound **4,** bearing a 3,4‐dichlorophenyl moiety, shows MIC values consistently lower than those reported for compound **3** for the same strains, reaching very low values of 0.5–1 μg mL^−1^ on several isolates. Noteworthy is the activity improvement of novel derivative **4** compared to lead **1** against *S. aureus* 18, *S. epidermidis 22*, and *S. epidermidis 2 R* (MIC values of 2 μg mL^−1^ compared to 4 μg mL^−1^ of lead compound **1**), whereas activity against *S. faecalis* and *S. faecium* remained unchanged.^[^ ^3^ ^]^

**Figure 2:**
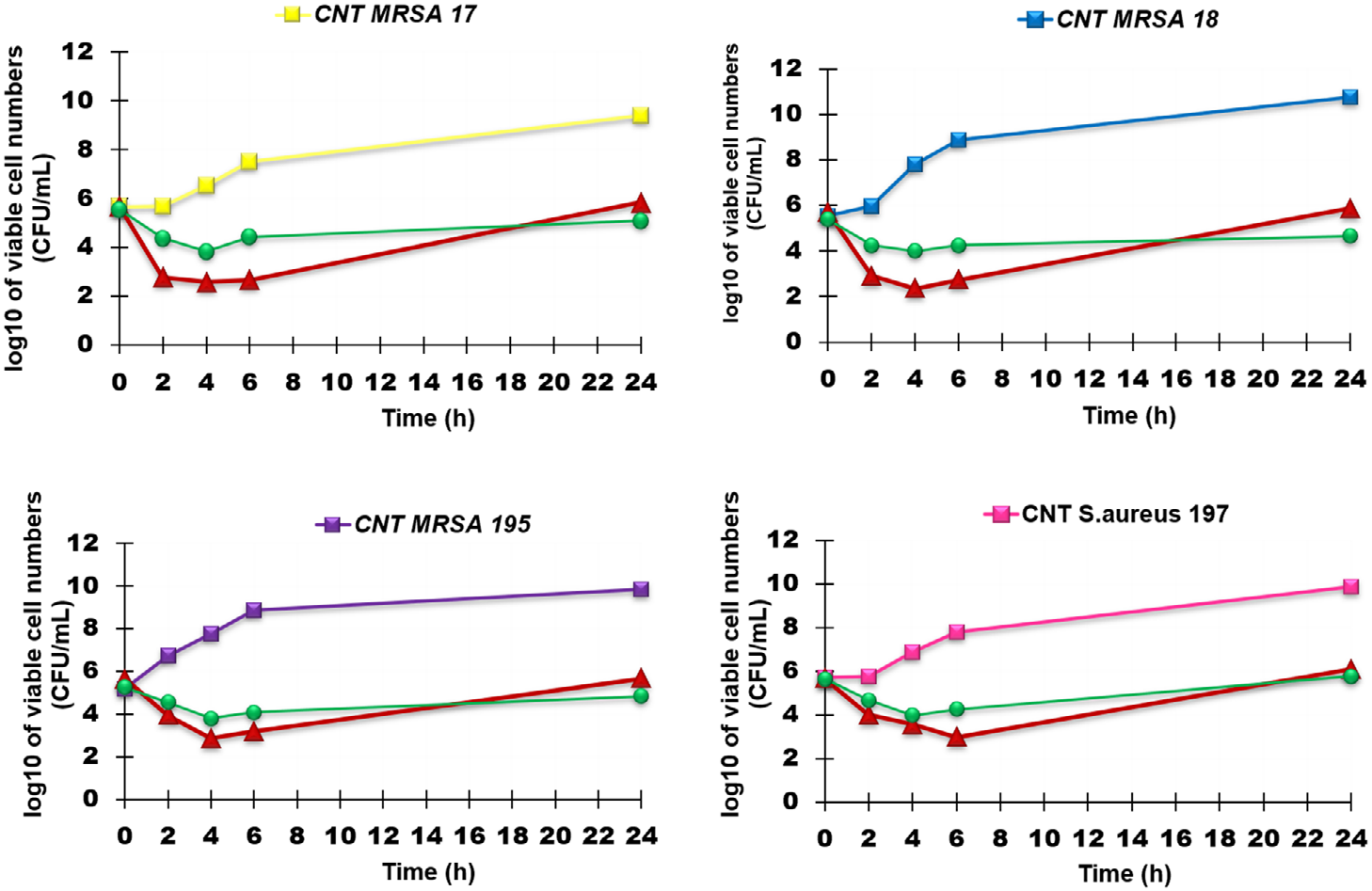
Time‐killing curves performed with compounds **3** (in red) and **4** (in green) at concentrations of 4x MIC on three MRSA strains *(*17,18, 195) and *S. aureus* 197. Untreated control strains were run in parallel (CNT). CNT = control.

To ascertain the nature of the antibacterial activity exhibited by compounds **3** and **4**, i.e., whether it is bacteriostatic or bactericidal, time‐killing experiments were conducted on four of the *S. aureus* strains used in this study. These experiments were conducted at concentrations of 4x the MIC. Similar results were obtained for all the isolates (**Figure** **2**), clearly indicating that both compounds are bacteriostatic. Indeed, both molecules, but particularly compound **3**, lowered the concentration of the initial inoculum for the first few hours of the experiment and limited its subsequent increase over the following 24 h. It appears that compound **4** was slightly more effective in its bacteriostatic action at 24 h, allowing for the reduction of the initial inoculum by approximately one order of magnitude.

### 2.3 Cytotoxicity Evaluation

**Figure 3:**
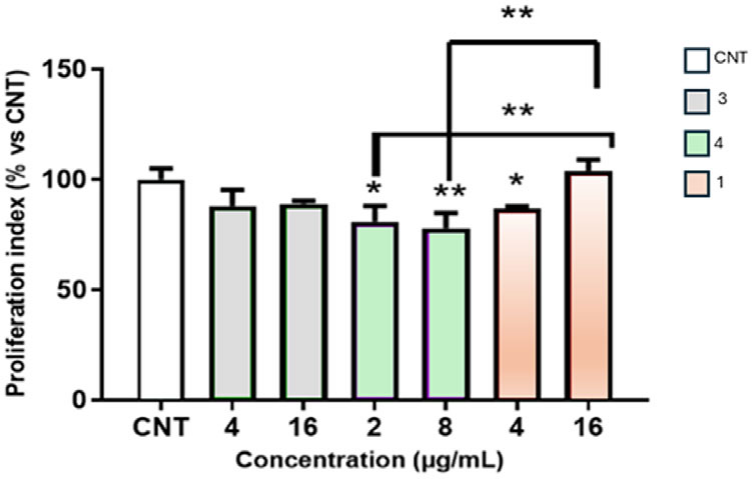
Cytotoxicity of **1**, **3,** and **4** at different selected concentrations toward Vero cells after 24 h. Data, expressed as percentage of viability versus untreated cultures (CNT) and extrapolated by MTT assay, are means ± SD of 3 separate experiments run in triplicate. * and ** significant difference (*p* < 0.05 and 0.01) versus CNT (one‐way ANOVA and Bonferroni test). SD = standard deviation; ANOVA = analysis of variance.

To verify if pyrazolines **1**, **3**, and **4** have any cytotoxic activity, we tested all three derivatives on Vero cells at the most representative MIC values obtained, i.e., at the MIC and 4x MIC concentrations (**Figure** **3**).

All tested concentrations showed no significant toxic effects on cell vitality, extrapolated by 3‐(4,5‐dimethylthiazol‐2‐yl)‐2,5‐diphenyltetrazolium bromide (MTT) index as shown in Figure 3. Both tested concentrations of **3**, i.e., 4 and 16 μg mL^−1^, resulted in a similar decrease in the cell proliferation index after 24 h of exposure. For compound **4**, the MTT test showed a reduction in the cell proliferation index of 81% and 78% after a 24 h exposure to concentrations of 2 and 8 μg mL^−1^, respectively. Compound **1** led to an initial 87% reduction in the cell proliferation index at a concentration of 4 μg mL^−1^, while at a concentration of 16 μg mL^−1^ it caused an increase of 104% in the cell proliferation index after 24 h of treatment.

Based on these preliminary cytotoxicity results and according to the ECVAM guidelines (which indicate that a chemical compound is considered toxic if cell viability is reduced by 15% compared to untreated cultures), it can be concluded that the tested samples do not significantly compromise the mitochondrial compartment of the cell tested, since the MTT test did not highlight toxic effects. It is noteworthy that compound **1** appears to give an increased cell proliferation index at a concentration of 16 μg mL^−1^. Further and more in‐depth studies on different cell lines will be necessary to confirm these interesting results.

### 2.4 Pharmacokinetic Properties, Drug‐Likeness, and Toxicity Prediction

**Figure 4:**
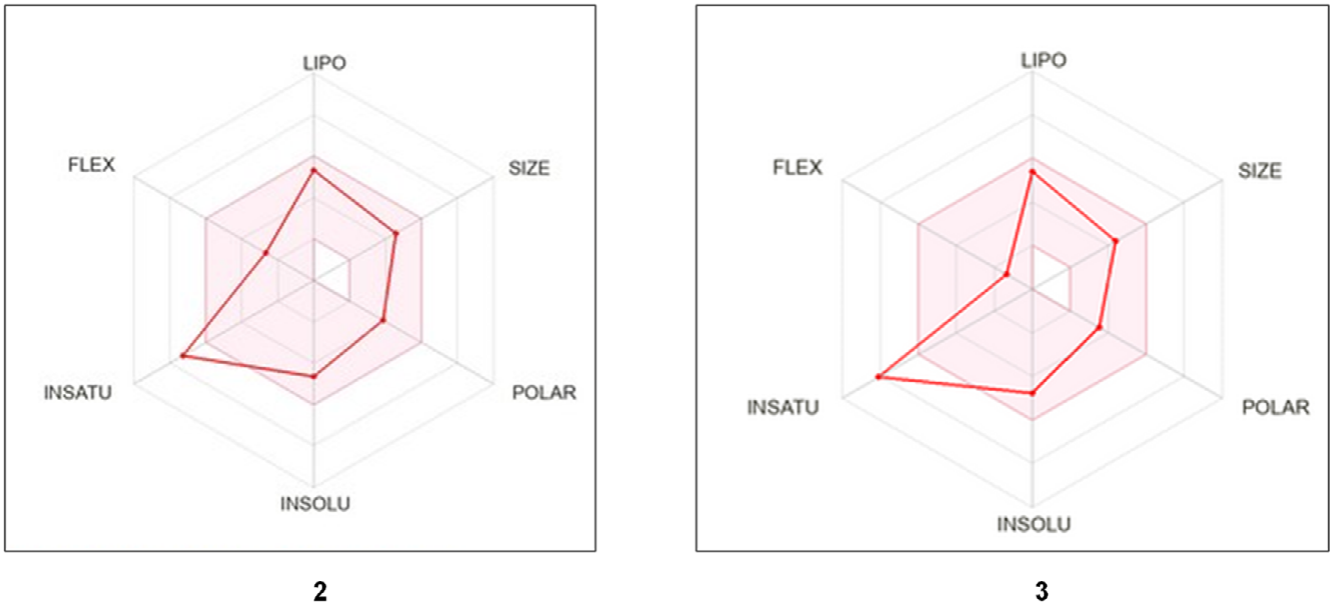
Radar plot calculated for compounds **3** and **4**. The pink area represents the optimal range for each property (lipophilicity, size, polarity, solubility, saturation, flexibility).

The pharmaceutical relevance of compounds **3** and **4**, their pharmacokinetics properties, as well as their drug‐likeness, were calculated using SwissADME^[^ ^8^ ^]^ and compared with data of derivative **1** from our previous report.^[^ ^3^ ^]^ The results are reported in **Table** **3** and **Figure** **4**.

**Table 3:** Predicted properties of bicyclic pyrazoline compounds 1, 3, and 4.

	**3**	**4**	**1**
Physicochemical property			
Molecular weight (MW) [g mol^−1^]	375.30	360.19	333.38
Fraction Csp^3^	0.17	0.12	0.25
Rotatable bonds	4	2	3
H‐bond acceptors	7	3	3
H‐bond donors	1	1	1
TPSAa) [Å^2^]	71.00	61.77	61.77
Lipophilicity			
LogPb)	3.84	3.92	3.79
Water solubility			
Solubility [mg mL^−1^]c)	8.38 × 10^−3^	5.97 × 10^−3^	1.18 × 10^3^
Solubility	Moderately soluble	Moderately soluble	Moderately soluble
Pharmacokinetics			
GI absorption	High	High	High
BBB permeant	Yes	Yes	Yes
Pgp substrate	No	No	No
CYP1A2 inhibitor	No	No	No
CYP2C19 inhibitor	Yes	Yes	Yes
CYP2C9 inhibitor	Yes	Yes	Yes
CYP2D6 inhibitor	No	No	No
CYP3A4 inhibitor	No	No	Yes
Drug‐likeness			
Lipinski violations	0	0	0
Medicinal chemistry			
PAINS alerts	0	0	0
Brenk alerts	1	1	1
	Phthalimide fraction	Phthalimide fraction	Phthalimide fraction

a) Topological polar surface area.

b) Predicted according to XLOGP3 program.

c) Values predicted by ESOL method.

In detail, good drug‐likeness and pharmacokinetic properties were predicted for the tested compounds, particularly regarding physicochemical properties, lipophilicity and water solubility. Specifically, their LogP values are 3.84 and 3.92, the number of rotatable bonds is 4 and 2, the number of H bond acceptors is 7 and 3, the number of H‐bond donors is 1, and the topological polar surface area (TPSA) is 71 and 61.77 Å^2^. The TPSA value of derivative **4** is the same as that of the previously studied compound **1**, and taken together these compounds are predicted to have a good ability to permeate membrane cells. Compounds **1**, **3**, and **4** are predicted to be moderately soluble and appear to be able to permeate the blood–brain barrier (BBB) without being substrates for Pgp. At the same time, permeation of the gastrointestinal (GI) tract is predicted as good for all reported compounds. In addition, no violations of the Lipinski rules were detected, and neither were any pan assay interfence compounds (PAINS) alerts found.

According to the calculations, and contrasting compound **1**, compounds **3** and **4** are unlikely to inhibit cytochrome (CYP) isoform 3A4, whereas the profile of inhibition of other isoforms (1A2, 2C19, 2C9, 2D6) is the same for all these three derivatives. Finally, as previously noticed for compound **1**, phthalimide fraction is considered problematic by Brenk filter indicating a possible point of chemical instability.

Taking all this information together, derivatives **3** and **4** are therefore characterized by good physiochemical, pharmacokinetic, and drug‐likeness properties, while Csp^[^ ^3^ ^]^ values below 0.25 are observed (0.17 for derivative **3** and 0.12 for derivative **4**, Table 3 and Figure 4).

Finally, the toxicity profiles of pyrazolines **1**, **3**, and **4** were predicted using the ProTox webserver.^[^ ^9^ ^]^ According to the simulation, all three pyrazolines are predicted to belong to toxicity class 4; in detail, the predicted LD_50_ value for derivatives **1** and **3** is 750 mg kg^−1^, and for derivative **4** 900 mg kg^−1^. None of these derivatives indicate signs of hepatotoxicity, cardiotoxicity, and nephrotoxicity, while they may have neurotoxic properties due to being able to cross the BBB.^[^ ^10^ ^]^ Lastly, no toxicity target pharmacophores have been detected (Novartis off‐targets, adenosine A2a receptor, adrenergic β 2 receptor, androgen receptor, amine oxidase A, corticotropin‐releasing hormone receptor 1, dopamine D3 receptor, estrogen receptor 1, estrogen receptor 2, glucocorticoid receptor, histamine H1 receptor, nuclear receptor subfamily 1 group I member 2, opioid receptor κ 1, progesterone receptor, phosphodiesterase 4D, prostaglandin G/H synthase 1).

## 3 Conclusion

With the aim to expand structure–activity relationships (SARs) of a new class of recently reported entities, new bicyclic pyrazolines decorated with functionalized phenyl pyrazoline moieties have been synthesized in continuous flow mode and evaluated for their antimicrobial activity. From these investigations, pyrazolines **3** and **4** emerged as potent bacteriostatic agents able to target different MDR strains of the *Staphylococcus* and *Enterococcus* genera with MIC values ranging from 0.5 to 4 μg mL^−1^, which in some cases are lower than those of the initial hit compound **1**. In addition, their cytotoxic evaluation on Vero cells (MTT test) highlighted that none of these derivatives significantly compromises the mitochondrial compartment of the cell, resulting in no cytotoxicity at the concentrations corresponding to the MIC and 4x MIC values. In addition to having good physiochemical, pharmacokinetic, and drug‐likeness properties, in silico evaluations suggest that these pyrazolines are not toxic (predicted toxicity class 4). Although in‐depth SAR considerations cannot be drawn, it can be highlighted that the introduction of halogen atoms clearly improves antimicrobial activity, as well as the chemical–physical properties of the new compounds, since *in silico* studies predicted a better solubility (8.38 × 10^−3^ and 5.97 × 10^−3^ mg mL^−1^ of **3** and **4** with respect to 1.18 × 10^−3^ mg mL^−1^ for **1**) and an ameliorated CYP 3A4 inhibition profile for the novel compounds **3** and **4** with respect to the original lead compound **1**. Collectively, this preliminary investigation demonstrates that the novel pyrazolines reported here represent a promising starting point for the further development of this novel antibacterial chemotype for possible use against difficult to treat bacterial species. Additional studies are necessary to identify their exact targets and the associated mechanism of action.

## 4 Experimental Section

4.1

4.1.1

##### General Information: Materials and Synthetic Methods

Solvents were purchased from Sigma–Aldrich and Fisher Scientific and used without further purification. Substrates and reagents were purchased from Alfa Aesar, Fisher Scientific, Fluorochem, or Sigma–Aldrich, and used as received. ^1^H NMR spectra were recorded with 400 and 500 MHz instruments and were reported relative to residual solvent: CHCl_3_ (*δ* = 7.26 ppm). ^13^C NMR spectra were recorded with the same instruments (100 and 125 MHz) and again were reported relative to CHCl_3_ (*δ* = 77.16 ppm). Data reported for ^1^H NMR were as follows: chemical shift (*δ *ppm^−1^) (multiplicity, coupling constant [Hz], integration). Multiplicities were reported as follows: s = singlet, d = doublet, t = triplet, q = quartet, p = pentet, h = heptet, and m = multiplet. Data for ^13^C{^1^H} NMR were reported in terms of chemical shift (*δ* ppm^−1^) and multiplicity (C, CH, CH_2_, or CH_3_). COSY, HSQC, and HMBC experiments were used in the structural assignment. Infrared spectra were recorded with a Bruker Platinum spectrophotometer (neat, ATR sampling) with the intensities of the characteristic signals being reported as weak (w, <20% of the tallest signal), medium (m, 21%−70% of the tallest signal), or strong (s, >71% of the tallest signal). High‐resolution mass spectrometry was performed using the indicated techniques with a micromass LCT orthogonal time‐of‐flight (TOF) mass spectrometer with leucine–enkephalin (Tyr–Gly–Phe–Leu) as an internal lock mass. For UV/VIS measurements, a Shimadzu UV‐1800 UV spectrophotometer was used. Continuous‐flow experiments were performed with a Vapourtec E‐Series system equipped with a UV150 photoreactor in combination with a high‐power LED emitting light at 365 nm wavelength and a medium‐pressure Hg lamp (combined with a low‐pass filter).

##### Antimicrobial Activity: Bacterial Species Evaluated in this Study

All the 36 clinical strains employed in this study belonged to a collection of Gram‐positive and Gram‐negative species obtained from the School of Medicine and Pharmacy of the University of Genoa (Italy). All were clinical strains isolated from human specimens and identified by VITEK 2 (bioMérieux, Firenze, Italy) or the matrix‐assisted laser desorption/ionization (MALDI‐TOF) mass spectrometric technique (bioMérieux, Firenze, Italy). Of the thirty‐three Gram‐positive organisms tested, twenty‐five isolates belonged to the genus *Staphylococcus* and included five *Staphylococcus aureus* strains, four of which were resistant to methicillin (MRSA), five *Staphylococcus epidermidis* isolates, four of which were resistant to methicillin (MRSE) and one also to linezolid, and fifteen more isolates pertaining to eight different species including one *S. auricularis* (resistant to methicillin), three *Staphylococcus capitis* (two were resistant to methicillin), three *Staphylococcus haemolyticus* (two were resistant to methicillin), two *S. hominis* (both resistant to methicillin), two *Staphylococcus lugdunensis* (one was resistant to methicillin), one *S. saprophyticus*, two *Staphylococcus simulans* (both resistant to methicillin, and one *Staphylococcus warneri* (resistant to methicillin).The eight strains of the *Enterococcus* genus included four *Enterococcus faecalis* isolates, all VRE (two were also resistant to teicoplanin), and four *Enterococcus faecium* strains, three of which were VRE and one was also resistant to teicoplanin. Among the three Gram‐negative strains, one was an *Escherichia coli* producing New Delhi metallo‐β‐lactamase, one was an MDR isolate of *Pseudomonas aeruginosa,* and one was a *Klebsiella pneumoniae* strain producing *Klebsiella pneumoniae* carbapenemase.

##### Antibacterial Susceptibility Tests

Values of the MICs were determined following the microdilution procedures detailed by the European Committee on Antimicrobial Susceptibility Testing EUCAST,^[^ ^11^ ^]^ as also reported in our previous studies.^[^ ^12^ ^]^

Briefly, serial twofold dilutions in Mueller–Hinton (MH) broth (Merck, Darmstadt, Germany) of all the samples (dissolved in dimethyl sufoxide (DMSO)), ranging from 128 to 1 μg mL^−1^, were used. DMSO was also tested as control to verify the absence of antibacterial activity of the solvent used for the experiments. Bacterial cultures obtained after overnight incubation were diluted to yield a standardized inoculum of 1.5 × 10^8^ CFU mL^−1^. Appropriate aliquots of each suspension were added to 96‐well microplates containing dilutions of compounds to be tested to yield a final concentration of about 5 × 10^5^ cells mL^−1^. After 24 h of incubation at 37 °C, the lowest concentration of sample that prevented visible growth was recorded as the MIC. All MICs were obtained at least in triplicate; results were expressed reporting the modal value, that is the value that has been observed most frequently. In case of equivocal or not clear results, more than three determinations of MICs were carried out.

##### Time Killing Curves

Killing curve assays for compounds were performed on the four *S. aureus* isolates selected for the study as previously reported.^[^ ^12^ ^]^ Mid‐logarithmic phase bacterial cultures were diluted in MH broth (10 mL) containing 4x MIC of the two compounds to give a final inoculum of 5.0 × 10^5^ CFU mL^−1^. The same inoculum was added to MH broth, as a growth control. Tubes were incubated at 37 °C with constant shaking for 24 h. Samples of 0.20 mL from each tube were removed at 0, 2, 4, 6, and 24 h, diluted appropriately with a 0.9% sodium chloride solution to avoid carryover of compounds being tested, plated onto MH plates, and incubated for 24 h at 37 °C. Growth controls were run in parallel. The percentage of surviving bacterial cells was determined for each sampling time by comparing colony counts with those of standard dilutions of the growth control. Results have been expressed as log_10_ of viable cell numbers (CFU mL^−1^) of surviving bacterial cells over a 24 h period. All time‐kill curve experiments were performed in triplicate.

##### Cytotoxicity Evaluation: Maintenance of Cell Cultures

Vero cell line, derived from African green monkey kidney (*Cercopithecus aethiops*), was certified by STR DNA profile analysis by Biological Bank, a Core Facility of the IRCCS San Martino University Hospital‐IST National Institute for Cancer Research (Genoa, Italy). Cells were maintained in Dulbecco's modified Eagle's medium (Euroclone, Milan, Italy) supplemented with 10% fetal bovine serum and 2 mM of glutamine (Euroclone Milan, Italy), without antibiotics and cultured at 37 °C in a humidified incubator containing 5% CO_2_. When the original flask was ≈75% confluent, cells were subcultured by TrypLE Express (Invitrogen Life Technologies, Carlsbad, CA, USA) treatment and seeded in 96‐well plates at 10 × 10^3^ cells/well, 24 h before MTT assay.^[^ ^13^ ^]^ All cell cultures were found to be mycoplasma‐free during regular checks with the Reagent Set Mycoplasma Euroclone (Euroclone Milan, Italy).

##### MTT Assay

The three compounds (**1**, **3**, and **4**) were tested on Vero cells at different concentrations corresponding to the MIC and 4x MIC for each sample. Cell viability, in terms of cell proliferation index, was assessed using the MTT assay.^[^ ^13^ ^]^ The optical densities of the dissolved formazan crystals were determined spectrophotometrically at 570 nm. Results have been analyzed comparing treated cells with not treated control cells. A chemical compound was considered toxic if the cell viability was reduced by 15% compared to untreated cultures, according to the ECVAM's guidelines.

## Conflict of Interest

The authors declare no conflict of interest

## Supporting information

Supplementary Material

## Data Availability

The data that support the findings of this study are available in Supporting Information of this article.
