# Supplementary material for: Synthesis and Biological Evaluation of Bicyclic Pyrazolines with Promising Antimicrobial Activities
Source: ChemMedChem. 2025 May 27;20(14):e202500144. doi: 10.1002/cmdc.202500144 (PMC12276031; doi:10.1002/cmdc.202500144)
Supplement: Supplementary file 1 — Supplementary Material [file CMDC-20-e202500144-s001.pdf]

Supporting Information

Synthesis and Biological Evaluation of Bicyclic Pyrazolines with Promising Antimicrobial Activities

Debora Caviglia,[b,d] Anna Maria Schito,[b] Susanna Penco, [c] Chiara Brullo,*[d] and Marcus Baumann*[a]

Table of contents:

1. Materials and Methods SI-2

2. Synthetic Procedures SI-3

3. Copies of NMR Spectra of Compound **4** SI-8

1. Materials and Methods

Solvents were purchased from Sigma–Aldrich and Fisher Scientific and used without further purification. Substrates and reagents were purchased from Alfa Aesar, Fisher Scientific, Fluorochem, or Sigma–Aldrich, and used as received.

^{1}H NMR spectra were recorded with 400 and 500 MHz instruments and are reported relative to residual solvent: $CHCl_3$ (δ = 7.26 ppm). ^{13}C NMR spectra were recorded with the same instruments (100 and 125 MHz) and again are reported relative to $CHCl_3$ (δ = 77.16 ppm). Data reported for ^{1}H NMR are as follows: chemical shift (δ/ppm) (multiplicity, coupling constant (Hz), integration). Multiplicities are reported as follows: s = singlet, d = doublet, t = triplet, q = quartet, p = pentet, h = heptet, m = multiplet. Data for ^{13}C{^{1}H} NMR are reported in terms of chemical shift (δ/ppm) and multiplicity (C, CH, CH_2, or CH_3). COSY, HSQC and HMBC, experiments were used in the structural assignment.

IR spectra were recorded with a Bruker Platinum spectrophotometer (neat, ATR sampling) with the intensities of the characteristic signals being reported as weak (w, <20% of the tallest signal), medium (m, 21–70% of the tallest signal), or strong (s, >71% of the tallest signal).

High-resolution mass spectrometry (HRMS) was performed using the indicated techniques with a micromass LCT orthogonal time-of-flight mass spectrometer with leucine-enkephalin (Tyr-Gly-Phe-Leu) as an internal lock mass.

For **UV/Vis measurements**, a Shimadzu UV-1800 UV spectrophotometer was used.

Continuous-flow experiments were performed with a Vapourtec E-Series system equipped with a UV150 photoreactor in combination with a high-power LED emitting light at 365 nm wavelength and a medium-pressure Hg-lamp (combined with a low-pass filter).

2. Synthetic Procedures

Synthesis of tetrazole building blocks:

Following a previously reported method [*SynOpen* **2023**; *07*(01): 69-75; DOI: 10.1055/a-1995-1859], tetrazole building blocks were generated in batch mode analogously:

Step 1: To a solution of concentrated HCl (3 mL, 12 M) at 0 °C was added the desired aniline (12 mmol) under stirring. After 5 min, a solution of $NaNO_2$ (12 mmol in 4 mL water) was added slowly and the suspension was stirred for a further 10 min before a solution of $NaBF_4$ (20 mmol in 4 mL water) was added. The mixture was stirred for 10 min and then the solid diazonium product was isolated by filtration, washed with dilute NaBF4 solution (ca. 5% w/w), and dried under suction.

Step 2: To a suspension of benzamidine hydrochloride (1; 1 equiv., 0.4 M) and K_2CO_3 (3 equiv.) in MeCN/water (50:50) was added the diazonium tetrafluoroborate salt 2 (1 equiv.) at 0 °C. The mixture was stirred for 5 h at rt and then the aryl iminotriazine adduct was isolated as a yellow solid by filtration, washed with water, and dried under suction.

Step 3: A suspension of molecular iodine (1.2 equiv.) and KI (1.5 equiv.) was prepared in DMSO (0.2 M) and stirred for 10 min at rt. Solid K_2CO_3 (3 equiv.) and the aryl iminotriazine adduct (1 equiv.) were added and then the resulting mixture was heated to 100 °C for 1 h. After cooling to rt, the reaction mixture was quenched by addition of aq. $Na_2S_2O_3$, followed by extractive workup with EtOAc and aqueous brine. Purification by chromatography (silica gel, EtOAc/cyclohexane 3:97) gave the tetrazole product typically as a yellow oil or an off-white solid.

Spectroscopic data for the tetrazoles bearing a para-isopropyl group or para-OCF_3 group matched those published previously (see: *SynOpen* **2023**; *07*(01): 69-75; DOI: 10.1055/a-1995-1859) which is summarised below:

2-(4-Isopropylphenyl)-5-phenyl-2*H*-tetrazole:

Appearance: Yellow oil.

IR (neat): 2961 (m), 2870 (w), 1529 (m), 1511 (s), 1465 (s), 1449 (s), 1209 (s), 1052 (s), 835 (s), 729 (s), 689 (s) cm^{-1}.

^{1}H NMR ($CDCl_3$, 500 MHz): δ = 8.26 (dd, J = 8.0, 1.8 Hz, 2 H), 8.10 (d, J = 8.7 Hz, 2 H), 7.54–7.49 (m, 3 H), 7.42 (d, J = 8.4 Hz, 2 H), 3.02 (hept, J = 6.9 Hz, 1 H), 1.31 (d, J = 6.9 Hz, 6 H).

^{13}C NMR ($CDCl_3$, 125 MHz): δ = 165.0 (C), 150.8 (C), 134.9 (C), 130.4 (CH), 128.9 (2 CH), 127.6 (2 CH), 127.3 (C), 127.0 (2 CH), 119.9 (2 CH), 33.9 (CH), 23.9 (2 CH_3).

HRMS (ESI+): m/z [M + H]+ calcd for $C_{16}H_{17}N_4$: 265.1448; found: 265.1446.

5-Phenyl-2-(4-(trifluoromethoxy)phenyl)-2*H*-tetrazole:

Appearance: Off-white solid.

IR (neat): 3077 (w), 1610 (w), 1531 (m), 1263 (s), 1208 (s), 1179 (s), 1015 (m), 856 (m), 726 (s), 683 cm (s) $^{-1}$.

^{1}H NMR ($CDCl_3$, 400 MHz): δ = 8.26–8.19 (m, 4 H), 7.52–7.46 (m, 3 H), 7.41 (d, J = 8.0 Hz, 2 H).

^{13}C NMR ($CDCl_3$, 100 MHz): δ = 165.4 (C), 149.6 (C), 135.1 (C), 130.7 (CH), 129.0 (2 CH), 127.0 (2 CH), 126.8 (C), 122.1, 121.3 (2 CH), 120.3 (q, J = 257 Hz, CF_3).

^{19}F NMR ($CDCl_3$, 376 MHz): δ = –58.0 (s).

HRMS (ESI+): m/z $[M + H]^+$ calcd for $C_{14}H_{10}N_4OF_3$: 307.0801; found: 307.0802.

Crystal data (CCDC 2221466): P21/n; a 7.97598(18) Å, b 11.5500(2) Å, c 28.7873(7) Å, α = 90°, β = 93.383(2)°, γ = 90°.

2-(3,4-Dichlorophenyl)-5-phenyl-2*H*-tetrazole:

Appearance: Brown solid.

IR (neat): 3099 (w), 3035 (w), 1595 (m), 1529 (m), 1477 (s), 1449 (m), 1411 (m), 1211 (m), 1134 (m), 1019 (s), 814 (s), 726 (s), 687 (s) cm^{-1}.

^{1}H NMR ($CDCl_3$, 400 MHz): δ = 8.32 (d, *J* = 2.5 Hz, 1H), 8.23 – 8.19 (m, 2H), 8.05 (dd, *J* = 8.8, 2.5 Hz, 1H), 7.63 (d, *J* = 8.7 Hz, 1H), 7.54 – 7.48 (m, 3H).

^{13}C NMR ($CDCl_3$, 100 MHz): δ = 165.5 (C), 135.7 (C), 134.0 (C), 133.8 (C), 131.4 (CH), 130.8 (CH), 129.0 (2CH), 127.1 (2CH), 126.6 (C), 121.6 (CH), 118.7 (CH).

HRMS (ESI+): m/z $[M + H]^+$ calcd for $C_{13}H_9Cl_2N_4$: 291.0199; found: 291.0199.

Continuous Flow Synthesis of Bicyclic Pyrazolines

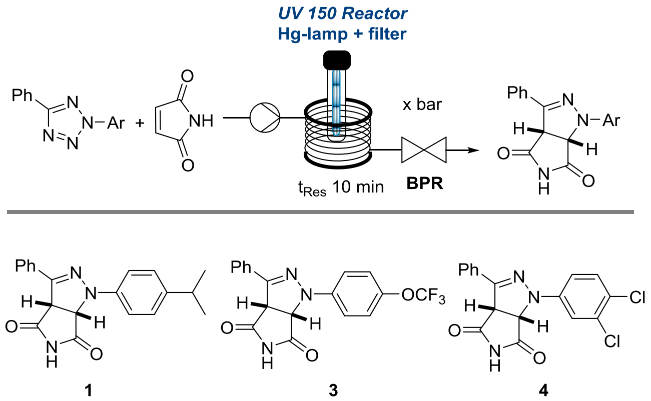

Procedure: In analogy to the published procedure [*Int. J. Mol. Sci.* **2023**, *24*(6), 5319] a homogeneous solution containing the tetrazole substrate (1 equiv.) and maleimide as the dipolarophile (1.2 equiv.) was prepared in MeCN (100 mM) and passed through the UV150 photoreactor of a Vapourtec E-Series system equipped with a medium-pressure Hg-lamp (85% input power, low-pass filter) and a flow coil (10 mL, PFA, residence time 10 min). Temperature control was provided via a stream of compressed air (ca. 28 °C internal reactor temperature). The exiting reaction mixture passed a BPR set to 2 bar before being collected in a flask. Evaporation of the solvent was followed by chromatography (silica gel, EtOAc/cyclohexane (10:90 to 20:80) to give the pyrazoline products after final evaporation of all volatiles.

Data for product 1: Following the described procedure **1** was produced as previously accessed matching the published spectroscopic data [*Int. J. Mol. Sci.* **2023**, *24*(6), 5319; https://doi.org/10.3390/ijms24065319]:

1-(4-Isopropylphenyl)-3-phenyl-3a,6a-dihydropyrrolo [3,4-c]pyrazole-4,6(1*H*,5*H*)-dione, 1:

Appearance: Yellow solid. Yield: 81%.

^{1}H NMR ($CDCl_3$, 400 MHz): δ/ppm 8.43 (s, 1H), 7.99 (d, *J* = 7.1 Hz, 2H), 7.46 (d, *J* = 8.7 Hz, 1H), 7.44–7.36 (m, 3H), 7.20 (d, *J* = 8.6 Hz, 2H), 5.11 (d, *J* = 10.9 Hz, 1H), 4.85 (d, *J* = 10.9 Hz, 1H), 2.87 (hept, *J* = 6.9 Hz, 1H), 1.23 (d, *J* = 7.0 Hz, 6H).

^{13}C NMR ($CDCl_3$, 100 MHz): δ/ppm = 172.6 (C), 171.5 (C), 142.5 (C), 142.3 (C), 142.1 (C), 130.3 (C), 129.4 (CH), 128.6 (2CH), 127.1 (2CH), 127.0 (2CH), 114.4 (2CH), 66.9 (CH), 54.6 (CH), 33.4 (CH), 24.1 (CH_3), 24.1 (CH_3).

IR (neat): 3255 (broad), 2959 (m), 2869 (w), 1784 (s), 1610 (w), 1514 (s), 1381 (m), 1342 (m), 1207 (m), 1192 (m), 827 (m), 736 (m) cm^{-1}.

HRMS (ESI+): m/z $[M+H]^+$ calcd for $C_{20}H_{20}N_3O_2$: 334.1550; found: 334.1551.

Crystal data (CCDC-2221468): P21/c; a 17.3845(5) b 6.2983(2) c 15.8490(3), α = 90°, β = 100.537(2)°, γ = 90°.

<u>**Data for product 3:**</u> Following the described procedure **2** was produced as previously accessed matching the published spectroscopic data [*SynOpen* **2023**; *07*(01): 69-75; DOI: 10.1055/a-1995-1859]:

3-Phenyl-1-(4-(trifluoromethoxy)phenyl)-3a,6a-dihydropyrrolo[3,4-c]pyrazole-4,6(1*H*,5*H*)-dione:

Appearance: Beige solid. Yield: 77%.

IR (neat): 3203 (m), 3086 (w), 1774 (w), 1706 (s), 1508 (s), 1256 (s), 1200 (s), 1169 (s), 1090 (m), 1016 (m), 843 (m), 805 (m), 767 (m), 687 (m), 622 (m) cm^{-1}.

^{1}H NMR ($DMSO-d_6$, 400 MHz): δ = 11.87 (br s, 1 H), 8.04–7.90 (m, 2 H), 7.52–7.46 (m, 2 H), 7.45–7.39 (m, 3 H), 7.31 (d, J = 8.7 Hz, 2 H), 5.33 (d, J = 10.7 Hz, 1 H), 5.13 (d, J = 10.7 Hz, 1 H).

^{13}C NMR ($DMSO-d_6$, 100 MHz): δ = 175.4 (C), 174.2 (C), 145.5 (C), 144.0 (C), 142.3 (C, q, J = 2 Hz), 130.8 (C), 129.9 (CH), 128.9 (2 CH), 127.5 (2 CH), 122.5 (2 CH), 120.7 (CF_3, q, J = 254 Hz), 115.2 (2 CH), 67.0 (CH), 55.6 (CH).

^{19}F NMR ($DMSO-d_6$, 376 MHz): δ = –57.2 (s).

HRMS (ESI+): m/z $[M + H]^+$ calcd for $C_{18}H_{13}N_3O_3F_3$: 376.0904; found: 376.0903.

<u>**Data for product 4:**</u>

1-(3,4-Dichlorophenyl)-3-phenyl-3a,6a-dihydropyrrolo[3,4-c]pyrazole-4,6(1*H*,5*H*)-dione:

Appearance: Beige powder. Yield: 75% (540 mg, 1.51 mmol).

IR (neat): 3291 (m), 3087 (w), 1776 (m), 1709 (s), 1591 (m), 1480 (s), 1300 (m), 1194 (s), 1125 (s), 794 (s), 689 (s) cm^{-1}.

^{1}H NMR ($DMSO-d_6$, 400 MHz): δ = δ 11.90 (s, 1H), 7.96 (dd, *J* = 8.0, 1.7 Hz, 1H), 7.59 (d, *J* = 2.7 Hz, 1H), 7.53 (d, *J* = 9.0 Hz, 1H), 7.48 – 7.36 (m, 4H), 5.36 (d, *J* = 10.6 Hz, 1H), 5.15 (d, *J* = 10.6 Hz, 1H).

^{13}C NMR ($DMSO-d_6$, 125 MHz): δ = 175.2 (C), 174.0 (C), 146.3 (C), 144.5 (C), 131.9 (C), 131.2 (CH), 130.5 (CH), 130.2 (C), 129.0 (2CH), 127.7 (2CH), 122.1 (C), 115.4 (CH), 114.5 (CH), 66.6 (CH), 55.6 (CH).

HRMS (ESI+): m/z $[M + H]^+$ calcd for $C_{17}H_{12}Cl_2O_2N_4$: 360.0301; found: 360.0299.

3. Copies of NMR Spectra for Compound 4

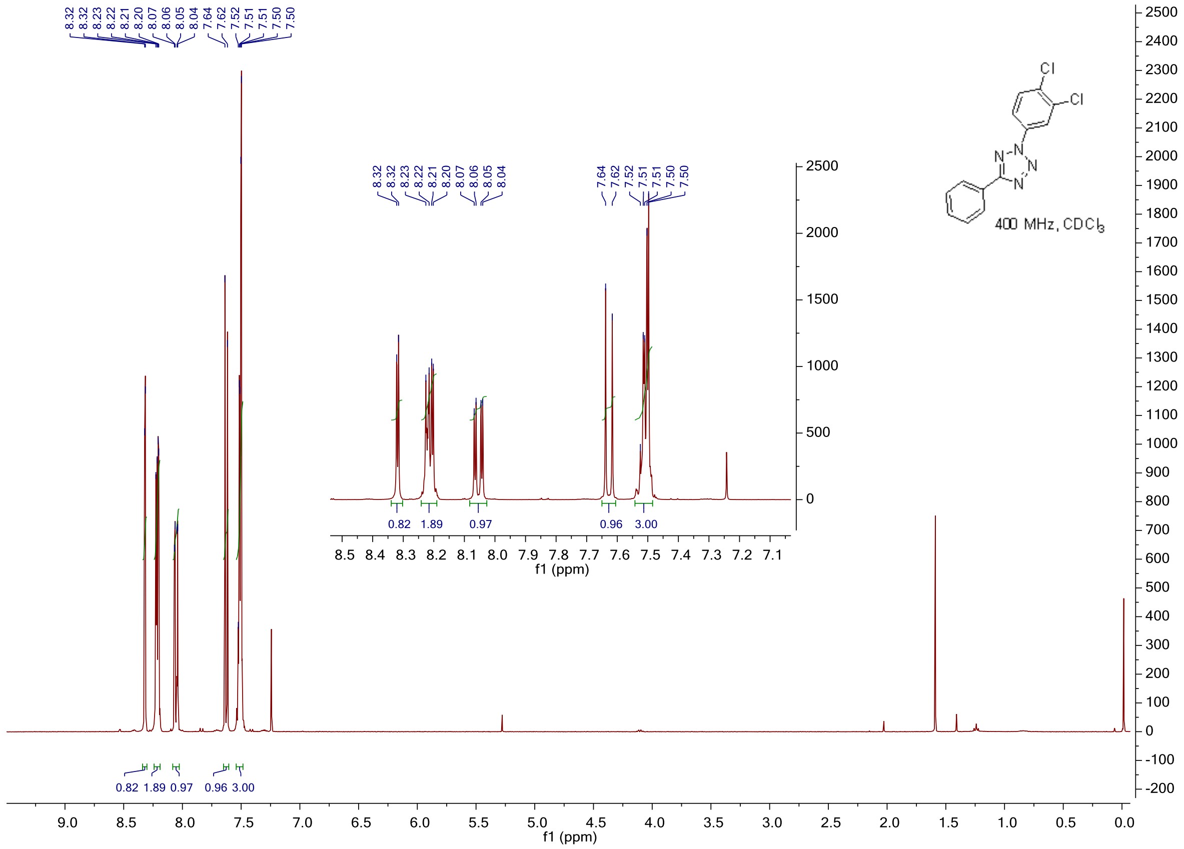

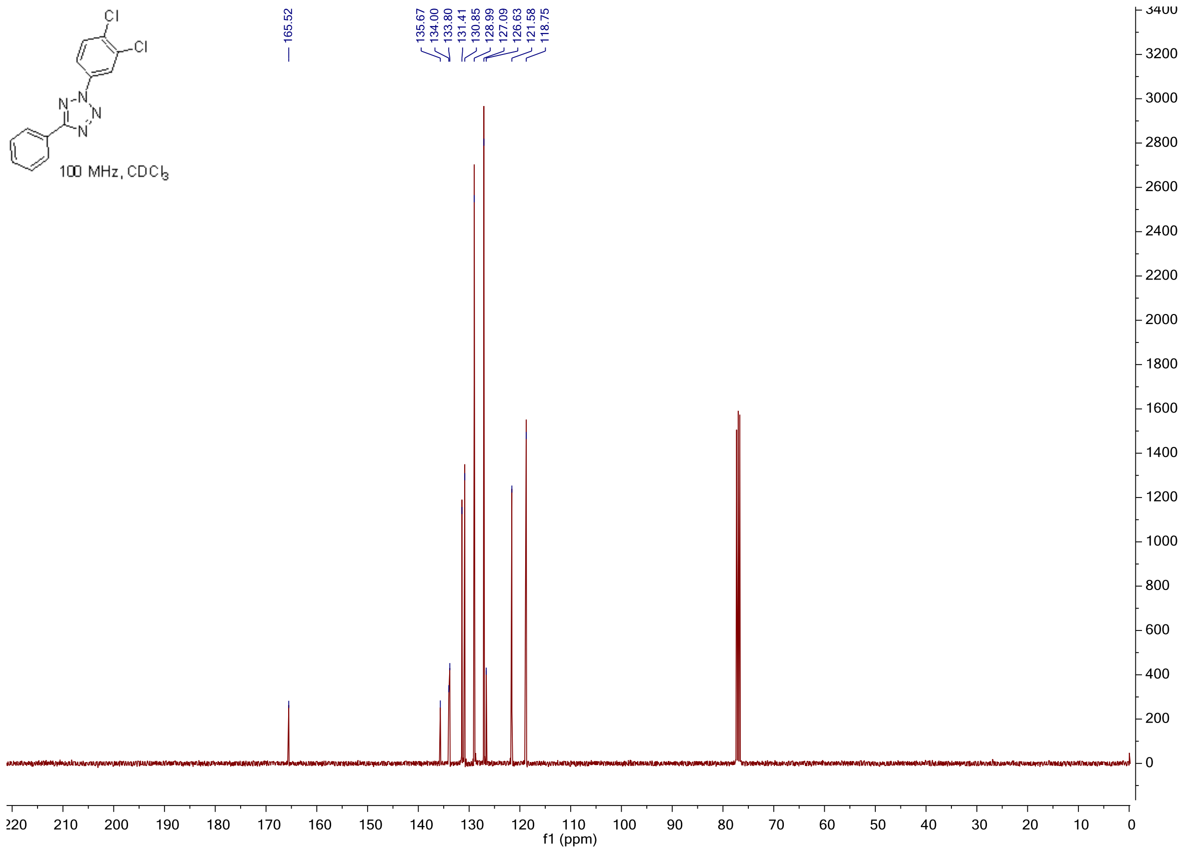

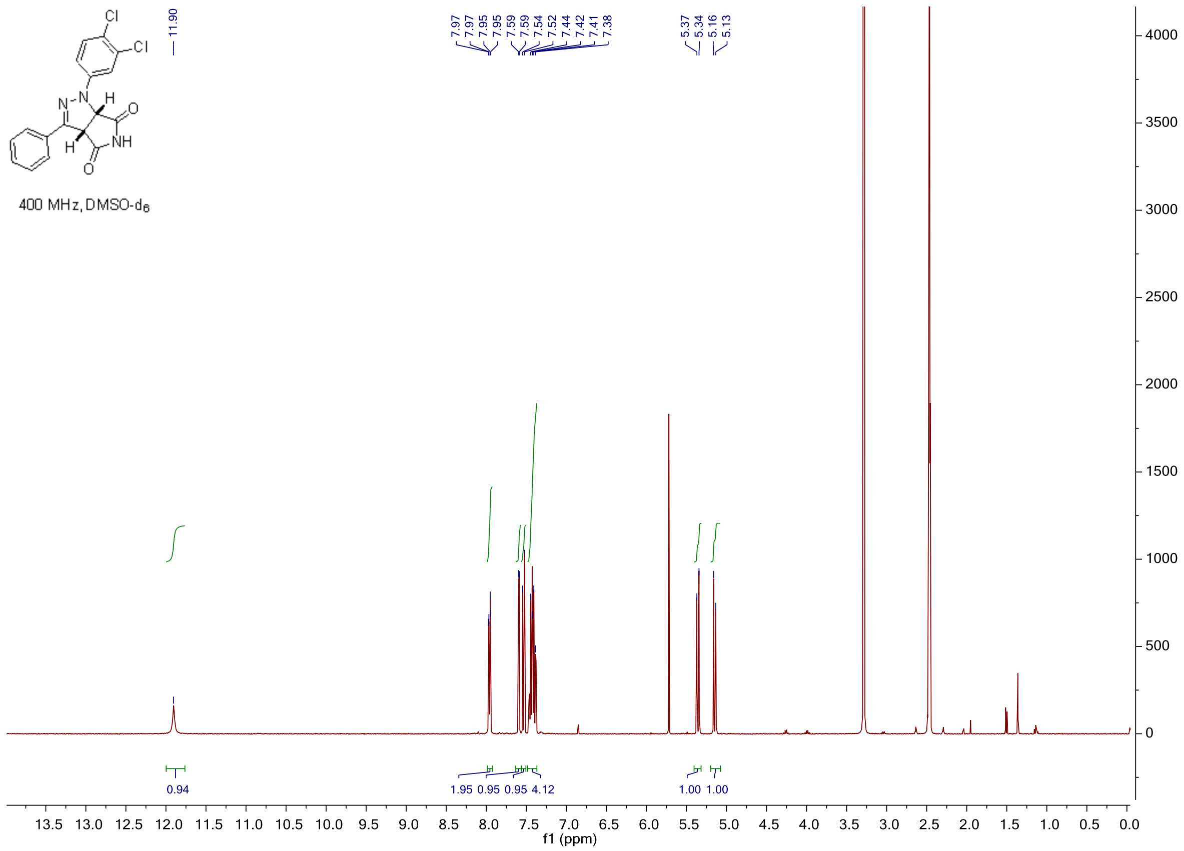
400 MHz, DMSO-d6
11.90
7.97
7.97
7.95
7.95
7.59
7.59
7.54
7.52
7.44
7.42
7.41
7.38
5.37
5.34
5.16
5.13
0.94
1.95 0.95 0.95 4.12
1.00 1.00
f1 (ppm)

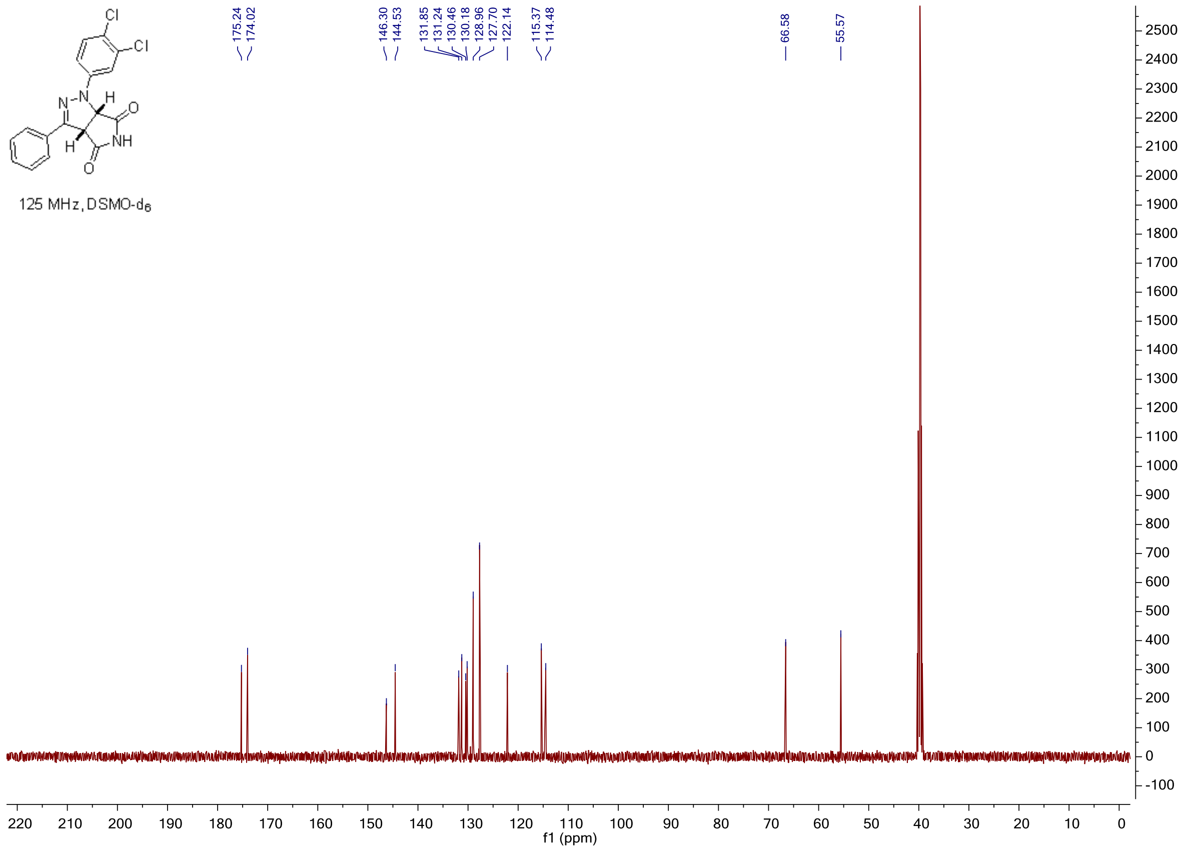
125 MHz, DSMO-d6
175.24
174.02
146.30
144.53
131.85
131.24
130.46
130.18
128.96
127.70
122.14
115.37
114.48
66.58
55.57
f1 (ppm)
